# The effect of age on discrimination learning and self-control in a marshmallow test for pigs

**DOI:** 10.1038/s41598-021-97770-x

**Published:** 2021-09-14

**Authors:** Annika Krause, Maren Kreiser, Birger Puppe, Armin Tuchscherer, Sandra Düpjan

**Affiliations:** 1 Institute of Behavioural Physiology, Research Institute for Farm Animal Biology (FBN), Wilhelm-Stahl-Allee 2, 18196 Dummerstorf, Germany; 2 Behavioural Sciences, Faculty of Agricultural and Environmental Sciences, University of Rostock, Rostock, Germany; 3 Institute of Genetics and Biometry, Research Institute for Farm Animal Biology (FBN), Dummerstorf, Germany

**Keywords:** Developmental biology, Psychology

## Abstract

Both humans and nonhuman animals need to show self-control and wait for a larger or better reward instead of a smaller or less preferred but instant reward on an everyday basis. We investigated whether this ability undergoes ontogenetic development in domestic pigs (similar to what is known in human infants) by testing if and for how long nine- and 16-week-old pigs wait for a larger amount of their preferred reward. In a delay-of-gratification task, animals first learned that a small reward was hidden under a white cup and a large reward under a black cup, and then the delay to deliver the large reward was gradually increased. The results show that older pigs could wait longer for a larger reward than younger pigs (10.6 ± 1.3 s vs. 5.2 ± 1.5 s), thereby confirming our hypothesis of ontogenetic development of self-control in pigs. This self-control is likely to be regulated by the behavioural inhibition system and associated systems. Self-control or, more specifically the lack of it may be involved in the development of abnormal behaviours, not only in humans but also in animals. Therefore, research on self-control in decision-making might provide a new perspective on abnormal behaviours in captive animals.

## Introduction

The everyday life of both humans and nonhuman animals is filled with conflicting motivations, forcing the individual to choose between two or more options on a regular basis. In his seminal study, Walter Mischel and colleagues^1^ investigated preschool children in a so-called delay-of-gratification task, offering them either one marshmallow there and then or two marshmallows after a waiting time. The question was whether the children could inhibit their initial impulse (i.e., to seek instant gratification and choose the immediate but less favourable reward) and wait for the second marshmallow (i.e., to accept the delay of gratification and choose the more favourable reward). This ability to suppress one motivation (e.g., for instant gratification) and withhold the associated behavioural response is called self-control (e.g. ref.^2^). Impulsivity, on the other hand, can be defined as ‘swift action without forethought or conscious judgment (1), behaviour without adequate thought (2), and the tendency to act with less forethought than do most individuals of equal ability and knowledge (3)^3^’ (see also ref.^4^ for a more detailed view on impulsivity). Impulsivity is in some ways considered the counterpart of self-control, and both can influence an individual’s decision-making (see ref.^2^, for an overview of relevant terms and concepts). As an old saying goes, ‘a bird in the hand is worth two in the bush’.

In the long run, however, this kind of delay discounting, i.e., the devaluation of (temporally) more distant rewards, can lead to maladaptive decision-making (see ref.^5^, for a formal model of when impulsivity can be adaptive). Mischel and colleagues^6^ have shown that those who had a high level of self-control as pre-schoolers did (on average) reach a higher level of education as well as a higher income and were healthier^6^. This ability, however, relies on cognitive capacities that are subject to ontogenetic development^7^. Mischel and Metzner^8^ hypothesized that younger children might not discriminate between different delays (see ref.^9^ for a model based on time perception) and therefore only develop this ability until they are approximately 9 years old. In addition to developmental aspects, however, self-control is a highly individual trait, as is impulsivity. Swann and colleagues^10^ showed a link between measures of reward-delay impulsivity and impulsivity as a personality trait. Moreover, impulsivity has been described not only in the context of various psychiatric disorders (e.g. refs^11–13^) but also in relation to social behaviour (e.g. refs^14,15^).

The question is whether self-control and impulsivity follow similar rules and developmental stages in nonhuman animals. For example, in the wild, many species rely on resources that are distributed in patches instead of evenly spread out in time and space. Therefore, when foraging, they might have to learn to decide whether to stay at a site with poor quality or quantity of food, or move on and search for a better, richer feeding site (which they might not find). Domestic pigs fulfil the basic requirements for tests of self-control, such as the food-related delay-of-gratification task, as they have a proven preference for larger rewards^16^, have a concept of time, can anticipate long-term consequences of their actions^17^ and remember the what/where/which of events^18^. In the ‘Pig Gambling Task’, a variation of the Iowa gambling task for humans, pigs can suppress their spontaneous preference for large but infrequent rewards when they learn that small but frequent rewards are preferable (i.e., they have an overall higher payoff) (e.g., ref.^19^). In a proof of concept study, Zebunke and colleagues^20^ found that domestic pigs can learn to wait for a larger or better reward and therefore show self-control. We now investigated whether this ability undergoes ontogenetic development. Pigs are acknowledged as a species capable of complex cognitive tasks^21–24^ (see also ref.^25^ for practical implications), but they might need the opportunity to gather experience during their ontogeny to solve social and physical tasks^26,27^ (but see ref.^28^). Therefore, it can be hypothesized that self-control also undergoes ontogenetic development in young pigs. Compared to humans, domesticated pigs undergo a faster ontogenetic development. They are usually weaned at 3 to 5 weeks of age, and they reach puberty at approximately 6 to 7 months of age, so the differences found between pre-schoolers and pre-pubertal children could be reflected by differences within only a few weeks of life in piglets.

To support our hypothesis, we investigated self-control in German Landrace piglets aged approximately 9 (late post-weaning period) or 16 weeks (growing period) using a delay-of-gratification task. We investigated whether and for how long they would wait for a larger amount of their preferred reward and analyzed the results with regard to their age. We expected to find a higher level of self-control, as indicated by a higher maximum delay, in the older pigs.

## Results

### Preference test

**Figure 1:**
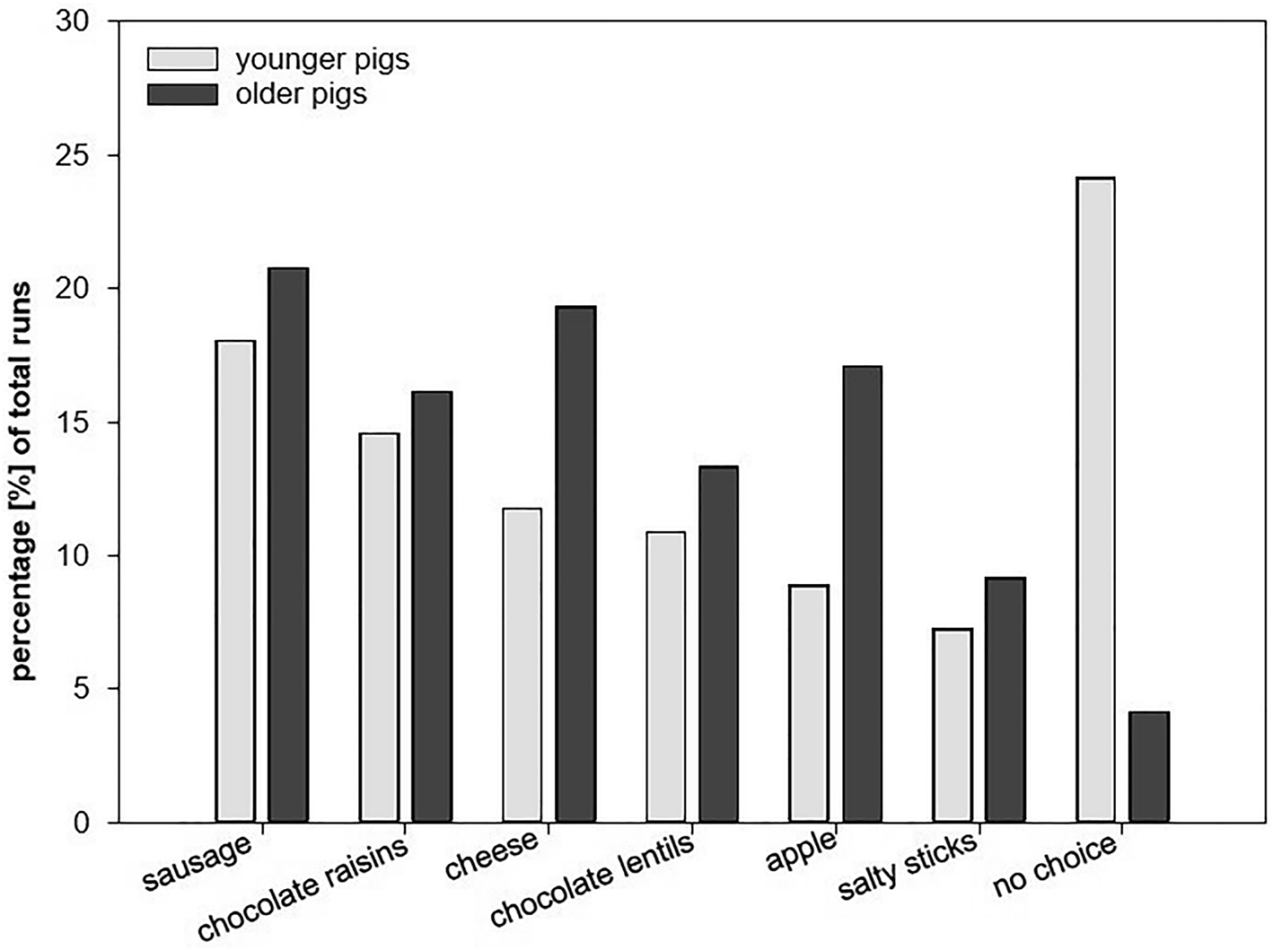
Percentage of food items chosen during the preference test with regard to the number of total runs per group (‘younger’ group: grey bars, N = 22 animals, total runs = 990; ‘older’ group: black bars, N = 23 animals, total runs = 1035).

Out of 2025 total runs, the pigs (*n* = 45) made 1732 choices (85.5%) and 293 omissions (14.5%) (all data in this section pooled from all pigs, individual differences are not accounted for). Regarding the two age-related groups, the ‘younger’ group (*n* = 22; 990 total runs) made 740 choices (74.4%) and 250 omissions (25.3%), while the ‘older’ group (*n* = 23; 1035 total runs) made 992 choices (95.8%) and 43 omissions (4.2%). Sausage was the most preferred food item in both age groups, followed by chocolate raisins, cheese, chocolate lentils, apple, salty sticks in the ‘younger’ group and cheese, apple, chocolate raisins, chocolate lentils, and salty sticks in the ‘older’ group (see Fig. 1).

### Discrimination task

**Figure 2:**
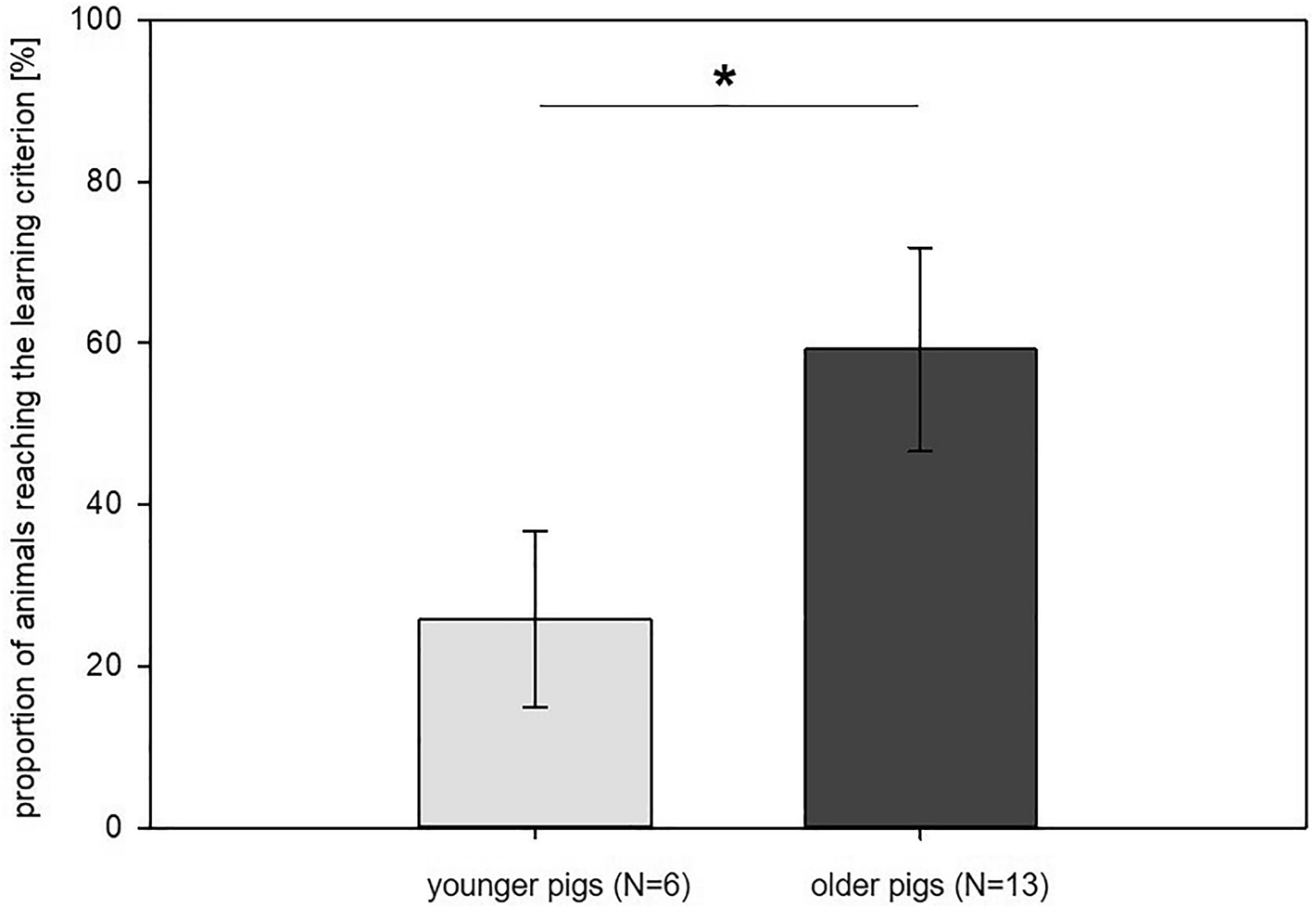
Performance of the animals in the discrimination task: Proportion of pigs in both groups (‘younger’ pigs, 7–8 weeks old at the time of testing vs. ‘older’ pigs: 14–15 weeks old at the time of testing) that reached the learning criterion during the training phase of 16 days. Data are presented as least squared means and standard errors (LSM ± SE); *p < 0.05.

A significant effect of age (‘younger’ group vs. ‘older’ group) was found on the performance of the pigs in the discrimination task (*F*_1,41_ = 4.22, *p* = 0.046). The proportion of pigs that reached the learning criterion in the discrimination task was significantly higher in the ‘older’ group than in the ‘younger’ group (see Fig. 2). Neither replicate (*F*_1,8.9_ = 0.24, *p* = 0.638) nor the interaction of replicate and age (*F*_1,41_ = 2.30, *p* = 0.137) was found to have an effect on the proportion of animals that reached the learning criterion.

The total number of sessions completed by the individual piglets during the discrimination task ranged from two to a maximum of 16 sessions in both age groups. In each age group, only one piglet needed two sessions to reach the learning criterion. Comparing the total number of sessions in those subjects that did reach the learning criterion, it emerged that there was no significant difference between age groups (*n* = 6, ‘younger’ group: 7.5 ± 1.6 sessions, *n* = 13, ‘older’ group: 8.9 ± 1.5 sessions, *F*_1,15_ = 0.71, *p* = 0.412).

### Delay-of-gratification task

**Figure 3:**
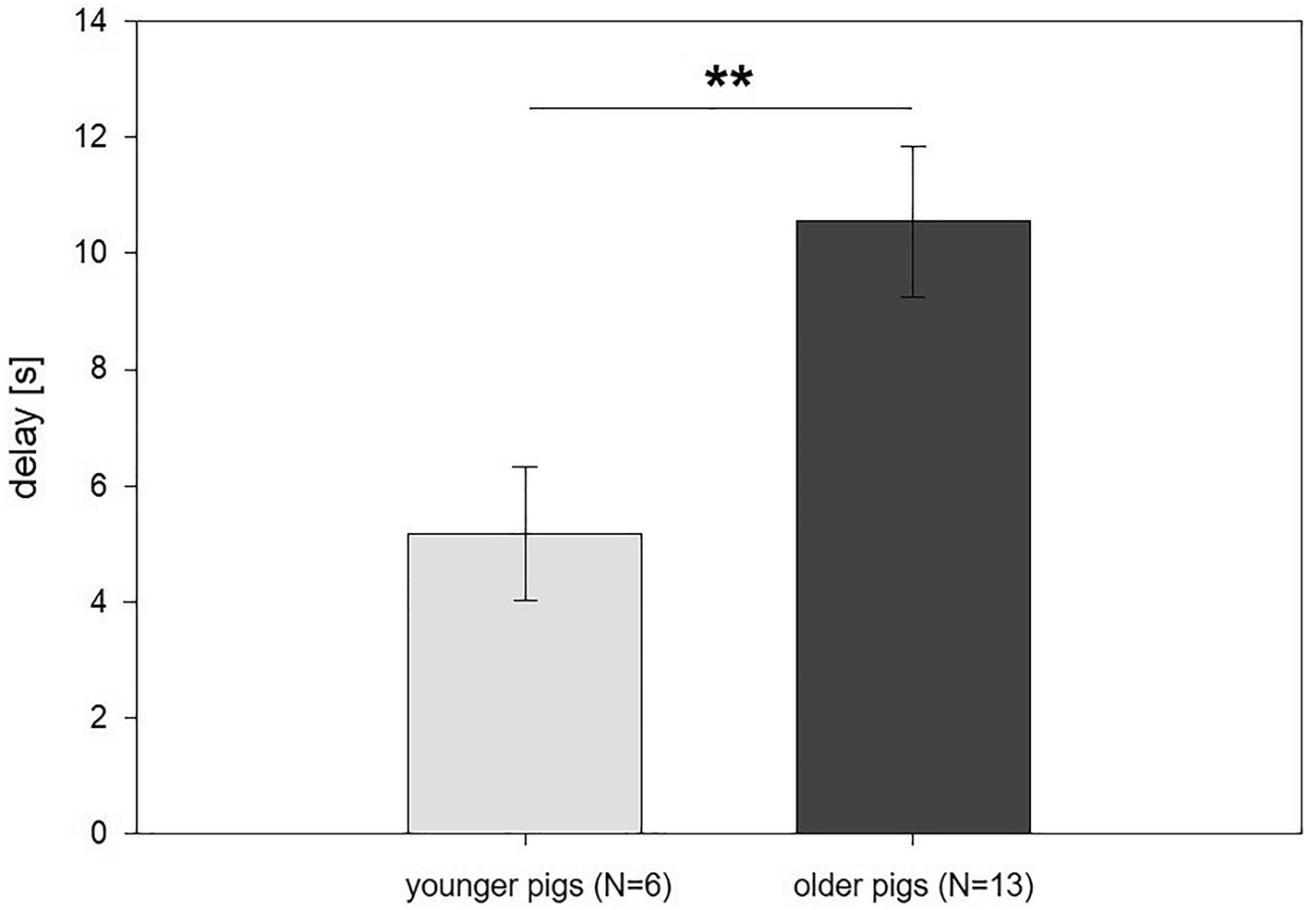
Performance of the animals in the delay-of-gratification task: Average maximum delay (in seconds [s]) mastered by the pigs in both groups (‘younger’ pigs: 7–9 weeks old at the time of testing vs. ‘older’ pigs: 14–16 weeks old at the time of testing) during the delay-of-gratification task. Data are presented as least squared means and standard errors (LSM ± SE);**p < 0.01.

The maximum delay reached by the piglets ranged from 4 s (that is, the piglets did master the first step of waiting 4 s for the larger reward) to the maximum delay step of 20 s mastered by one pig from the ‘older’ group. This means that no subject succeeded in the 26 s delay, and none reached testing for 32 s and 40 s. Comparing the maximum delay between both groups showed that the ‘older’ group mastered, on average, a maximum delay of 10.6 ± 1.3 s, while the ‘younger’ group mastered, on average, a delay of 5.2 ± 1.5 s. This age-related difference was found to be significant (*F*_1,15_ = 9.42, *p* = 0.008, see Fig. 3), whereas no effects of replicate (*F*_1,14.1_ = 0.01, *p* = 0.908) or the interaction of replicate and age (*F*_1,15_ = 0.05, *p* = 0.818) were found on the maximum delay.

## Discussion

We observed that older piglets were able to wait longer for a larger reward than their 7-week younger counterparts. In addition, a higher proportion of the older piglets learned the underlying discrimination task. Taken together, these results indicate the effects of ontogenetic development on the ability to show self-control in domestic pigs.

Using a delay-of-gratification paradigm, different age groups of pigs were tested for whether and for how long they would wait for a larger amount of their preferred reward instead of choosing the smaller, immediately available reward. As hypothesized, we found a relationship between the age of the pigs and their ability to show self-control, with pigs in the ‘older’ group reaching a higher maximum delay. This result might be surprising, given that the age difference was only 7 weeks. However, one must consider the faster ontogenetic development of domesticated pigs, which are usually weaned at 3 to 5 weeks of age (4 weeks in our experimental animals), and they reach puberty at approximately 6 to 7 months of age^29^, a month older than our ‘older’ subjects when tested in the delay-of-gratification task. Therefore, our results resemble findings in human infants, where self-control may start to show at 2 years of age (e.g. ref.^30^) but develops until the age of 9 years^8^ (see also ref.^31^).

Mischel and Metzner^8^ hypothesized that children need to develop the ability to perceive the differences in delays first, i.e., the ability to perceive time as a continuum (but see also ref.^9^ on how impulsivity can bias time perception). Špinka and colleagues^17^ investigated time perception in gilts, having them choose between a crate associated with 30-min short-term confinement or another crate associated with 4-h long-term confinement. The subjects demonstrated that they identified a difference between the two, meaning that they had a perception of time. Similarly, Fuhrer and Gygax^32^ found that dry sows can reliably estimate time in the range of days but not in the range of minutes. Compared to our experiment, however, these subjects were older, and the difference in time was larger. To our knowledge, there is only one study examining time perception in pigs^33^ in a range comparable to our study, where the subjects were supposed to press a lever for no less than 10 but no more than 14 s. They failed at this task, but not necessarily because they could not estimate the time, but because they could not handle the lever properly (attempting to press it with their feet instead of their snout) and therefore had poor control of how long it was pressed. Taken together, these results do not allow any conclusions regarding if and when time perception emerges in piglets’ ontogenetic development or if this ability is already present at birth.

Notably, the maximum delay reached by our subjects seems quite short. It is common, however, that while humans might report being willing to wait for months or even years, non-human animals mostly will wait for seconds only (e.g. ref.^34^). There are several potential factors influencing the degree of self-control (see ref.^34^, for a more detailed discussion), of which we want to mention the environment a species evolved in and the individual experiences. The feeding ecology of pigs is such that they have small food sources spread across their environment under natural conditions, which means that they do not usually have to wait for food for extended periods of time. While with the sows, piglets have to wait between nursing bouts, but there the situation is different. When wanting to drink, the piglets will massage their mother’s teats to induce milk flow, which gives them something to do in the waiting time. Even in humans, this kind of distraction was found to reduce delay discounting^1^. After weaning, domestic pigs (with the exception of breeding sows) are fed ad libitum and usually have permanent access to feed. Taken together, this means that our subjects had no prior experience with the delay of rewards they faced in our experiment. Therefore, the levels they reached were well within the expected range (see ref.^35^, Fig. 3, for an inter-species comparison).

Whether this kind of short-term delay discounting requires the full-blown understanding and perception of time that humans have, where time is linear with a past, future and present, and a linear sequence of events, is still under debate. While some authors consider animals as ‘stuck in time’ (e.g. refs^36,37^), and conclude that there is no conclusive evidence for mental time travel in non-human animals (e.g. refs^38,39^), others have challenged this idea (e.g. ref.^40^) based on experimental evidence for planning for the future in some primates and corvids. Delay of gratification, as tested in our study, does not necessarily require a linear time perception^37^, though, so our data support neither side.

However, although time perception is a necessary prerequisite for the delay-of-gratification task, it is not at the core of self-control per se. Self-control shows up in various situations, not only when choosing between immediate and delayed rewards but also whenever suppressing particular motivated behaviours is more adaptive. Central to the issue of self-control are neural pathways underlying motivation, decision-making, risk assessment and action control. Gray^41^ described the behavioural inhibition system (BIS), the behavioural activation system (BAS), and the fight-flight-freeze system (FFFS), which are relevant in this context (see ref.^42^, chapter 5, for the revised model as discussed in the following). The BIS regulates reward and punishment sensitivity systems and specifically moderates the FFFS, responsible for punishment avoidance, and the BAS, which activates behaviours to acquire rewards. In the case of the delay-of-gratification task, animals acquire rewards, while receiving a smaller reward can be considered a negative outcome, i.e., a punishment. This should result in the involvement of BIS, BAS and FFFS. BIS and BAS have been linked with impulsivity (e.g. refs^43,44^), personality (e.g. ref.^45^), and various psychiatric disorders (for review see ref.^46^). These are consistent individual traits, which can explain the significant individual variation in self-control found in our subjects (4 to 20 s). In humans, it has been shown that BIS scores increase during childhood and adolescence and reach a peak in early adulthood (e.g. ref.^47^). If pigs underwent similar temporal development, our subjects’ BIS should be in a phase of linear increase, resulting in significant differences between age groups, as seen in our data. To date, however, there is no BIS/BAS scale for pigs^48^. Given the evidence in humans, it is highly likely that BIS and BAS are involved in the regulation of self-control and that they link self-control and impulsivity with personality.

In humans, different brain networks have been associated with delay discounting, i.e., the devaluation of delayed rewards that counteracts self-control in a delay-of gratification task. These networks cover the processes of stimulus valuation and choice and are assumed to underlie delay discounting both as a trait and as a state (for review see ref.^49^). Top-down self-regulatory mechanisms might be reflected by changes in heart rate variability^50^, a physiological marker well established in domestic pigs^51^ and known to be related to individual adaptive strategies in affective responses to environmental challenges^52^. In this context, Beauchaine^53^ highlighted the important role of the autonomic nervous system and introduced an integrated model of autonomic nervous system-behaviour relations in which motivational functioning falls under sympathetic nervous system control and regulational functioning falls under parasympathetic nervous system control. This opens another approach to the investigation of mechanisms underlying self-control in pigs.

As a basis for the delay-of-gratification task, subjects in our study were required to learn stimulus-reward size contingencies in a visual discrimination task. Knowing which cup was associated with which reward size and delay would enable them to predict (future) outcomes of their actions and then show self-control in choosing to wait a certain period of time for the greatest payoff. Our findings have demonstrated that pigs can discriminate between magnitudes of rewards using visual cues (colour of the cup), even though vision is not their most well-developed sense (going back to ref.^54^; but see also refs^55–57^). Older pigs showed higher success in this visual discrimination paradigm than younger pigs, which is likely to be caused by differences in cognitive abilities rather than visual capacities. Cognitive abilities include the understanding of stimulus-reinforcer contingencies. There is, however, no experimental evidence that 5- to 6-week-old piglets are unable to learn such simple associations. In contrast, piglets of the same age can learn to associate spatial cues with rewards or punishments^58,59^, and they can even learn that the overall outcome of choosing small, but frequent rewards in the Pig Gambling Task is preferable to choosing large, but less frequent rewards in the long run^19^. Furthermore, we found no age difference in learning speed in those subjects who learned the discrimination task. Therefore, other aspects must underlie the age differences in the probability of learning. These are basically the same aspects underlying self-control, ranging from differences in food motivation and risk assessment to decision-making and action control. These are likely to be related to consistent individual traits (personality, temperament; see ref.^60^). One factor that did differ between age groups was the time they were housed in the experimental room. Therefore, the older groups had more time to habituate to the room and the presence of the experimenters. However, firstly, there was a habituation and handling phase for the younger groups, too, which was well within the range we usually do and find sufficient in our experiments. Secondly, we made an effort to minimize contact between experimenter and animals in the older pigs so that they experienced the actual training and testing, including the intensive handling by the experimenters and the experimental pen, as something new. Another potential difference between age groups was food motivation, as there were more omissions during the preference test in the ‘younger’ group than in the ‘older’ group. These tests were included based on our previous findings that pigs are willing to wait longer for their preferred reward than for a less preferred reward^20^ (see ref.^61^ for similar results in dogs). Our results clearly demonstrate that in pigs, as in humans, preferences for certain tastes vary among individuals. However, looking at the ranking of the different rewards in the two age groups, the preferences were quite similar between them, reflecting similar basic dietary requirements at this ontogenetic stage. And since we rewarded each subject with its individual, most preferred food reward as determined in the preference tests, we can dismiss food motivation as a cause for the differences found in the discrimination task and the delay-of-gratification task.

Self-control in domestic pigs is a relevant research object given the increasing use of pig models in biomedical research (e.g. ref.^62^). However, it is probably even more relevant for domestic pigs on farm, which show several abnormal behaviours that might be linked to or at least affected by deficits in self-control, similar to abnormal behaviours in human psychiatric disorders that are associated with self-control or impulsivity^49^ (see ref.^63^ for how a self-control technique could eliminate or reduce stereotypic behaviour in a multi-handicapped patient). However, in the literature, abnormal behaviours of captive animals are investigated with a focus on environmental factors both as the cause (e.g. refs^64,65^; but see also ref.^66^ for links between personality and abnormal behaviours in rhesus macaques) and as the cure (e.g. ref.^67^), while research on the regulatory systems underlying them is scarce^68,69^. Understanding self-control, how it develops, how it is linked to abnormal behaviours (especially injurious abnormal behaviours such as tail-biting (e.g. ref.^70^) and belly-nosing (e.g. ref.^71^)), and how it might be improved (comparable to self-control training in humans) can open new ways to reduce some of the most relevant problematic behaviours of pigs on-farm and animals in captivity in general.

To conclude, we found that self-control as tested in a delay-of-gratification task and learning performance in the underlying discrimination task are subject to ontogenetic changes. Older piglets showed more self-control, waiting longer for a larger reward. Future research should investigate the neurophysiological regulation of self-control, which we hypothesize lies within the behavioural activation system and behavioural inhibition system. This research approach might provide a new perspective on the occurrence of abnormal behaviours in captive animals.

## Animals, materials and methods

### Animals and housing

A total of 48 domestic pigs (*Sus scrofa*, German Landrace) from our institute’s experimental facilities for swine were used for this study in two replicates with 24 pigs each. Each replicate lasted 16 weeks, but the time each individual was tested depended on the group, individual learning speed and learning success and ranged from 2 to 5 weeks. In each replicate, the experimental pigs were recruited from six different litters raised in Scan farrowing pens. From every litter, four healthy, female, genetic full siblings were randomly selected. After weaning at 28 days of age, two groups with 12 piglets each were formed (‘older’ group and ‘younger’ group) that were housed in pens (3 m × 5 m) with partly slatted floors. They had ad libitum access to water and food provided at two troughs with six feeding places each. Straw and other physical enrichment were provided once a day. In addition to natural light, the lights were turned on from 0730 to 1600 h. Beforehand, a genetic analysis was performed to exclude pigs with a genotype that causes significant phenotypic deviations in plasma cortisol levels and adrenal weights^72^. One animal was excluded from further analysis due to medical treatment unrelated to our experiments.

### Experimental setup

**Figure 4:**
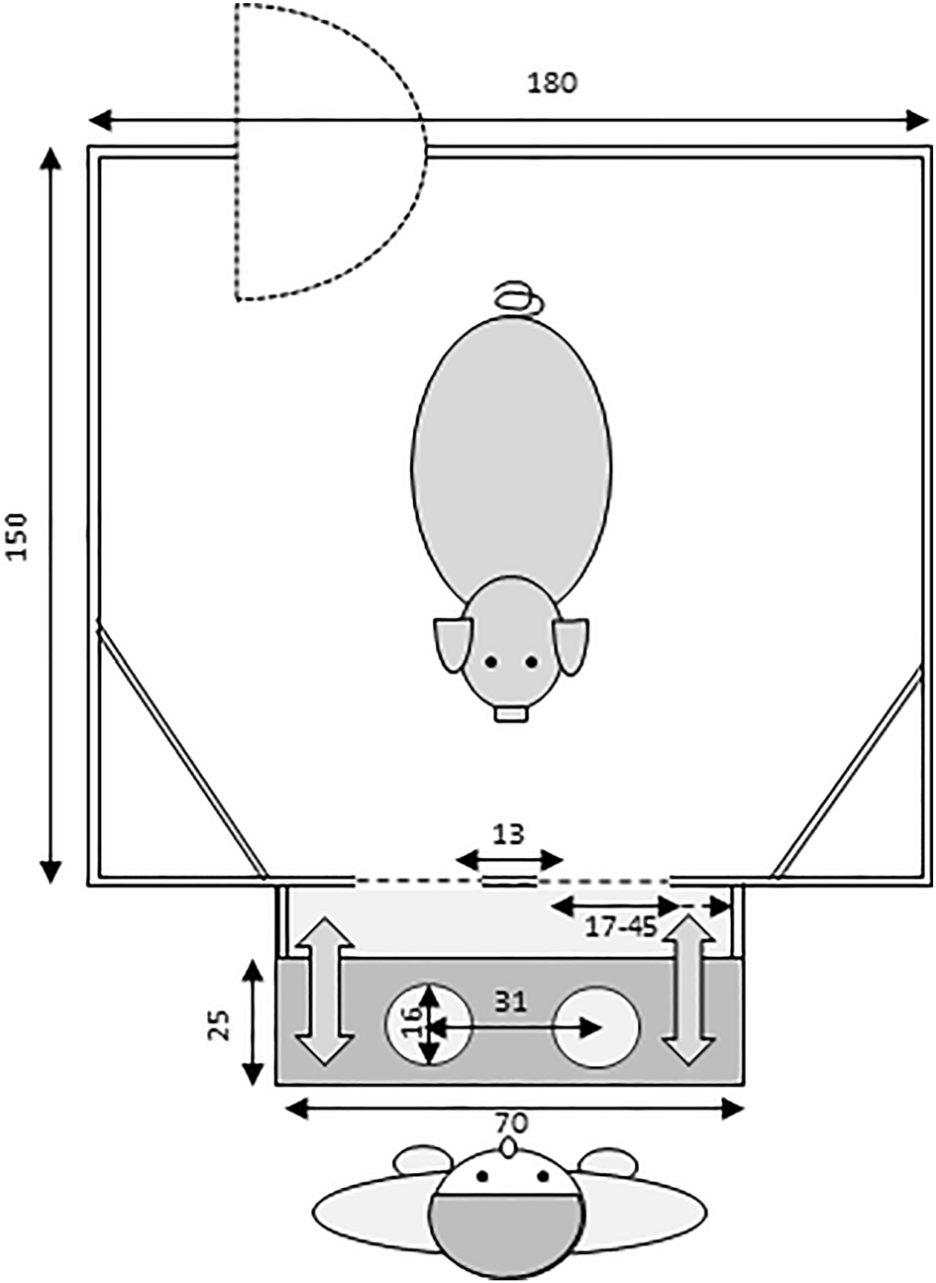
Top view sketch of the experimental pen (measurements given in cm).

The experimental pen (1.90 m × 1.5 m; see Fig. 4) was positioned in the same room as the pigs’ home pens, allowing for olfactory and auditory contact with the conspecifics. On one side of the experimental pen, two openings with adjustable widths were installed (15–45 cm, 13 cm apart from each other). The experimenter could sit directly behind these openings to handle a tray (70 cm × 25 cm) that could slide back and forth between the experimenter and subject. Two metal food bowls were placed on the wooden tray directly in front of the openings to present food rewards to the pig.

### Experimental procedure and data acquisition

The experiments were performed daily between 0800 and 1230 h. The two experimental groups started the experiment at either 5 or 12 weeks of age and will be referred to as ‘younger’ and ‘older’ throughout the manuscript. Their actual age changed throughout the experiment, and animals reached experimental stages at an individual pace.

**Figure 5:**
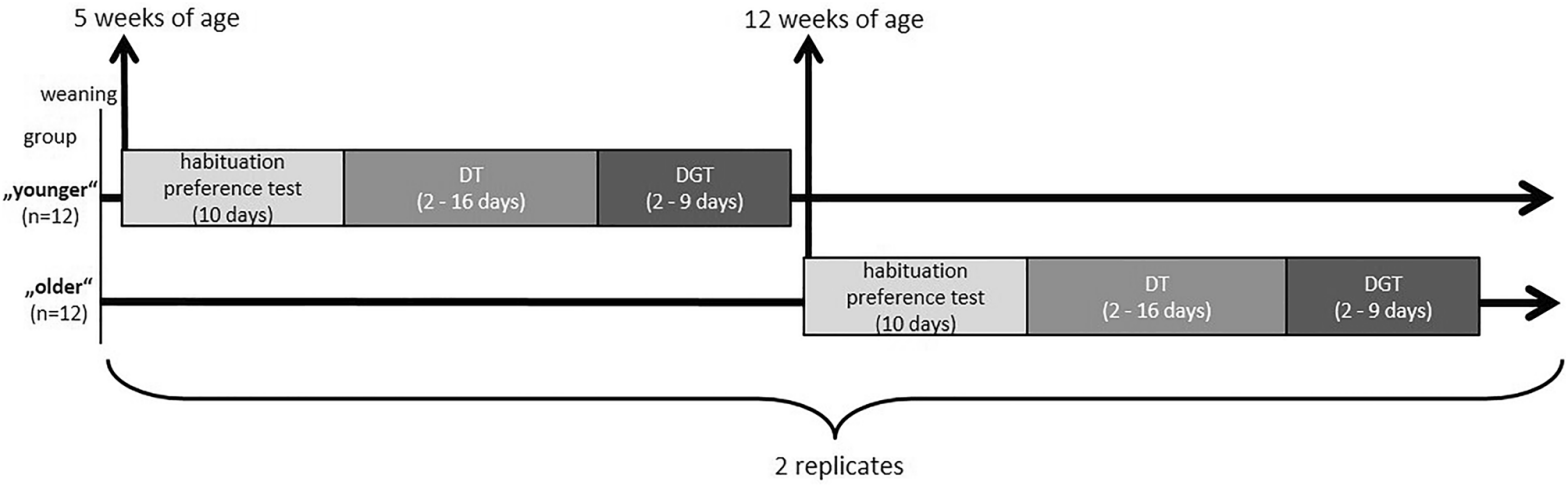
Schematic of the experimental timeline including habituation, the preference test, the discrimination task (DT) and the delay-of-gratification task (DGT) with two different age groups in two identical replicates.

The experiment was conducted in four phases: a habituation phase, a preference test, the discrimination task, and the delay-of-gratification task (Fig. 5).

#### Habituation

Four days after weaning, the habituation phase started with a stepwise accustomization to being handled, the experimental setup, including the experimental pen, the tray, the metal bowls, and the food rewards. Six different food rewards were used in this study: chocolate lentils, chocolate-coated raisins, apples, Gouda cheese, poultry sausages and salty sticks. The habituation phase lasted for 9 days and included two sessions per day on days one to four, which was reduced to only one session per day on days five to nine. On habituation days one and two, two randomly selected pairs of pigs explored the experimental pen for 10 min. The metal food bowls on the tray were filled with two pieces of each reward (= 12 pieces), and the tray was positioned in front of the openings such that the rewards were always within reach of the pigs. The shape of the openings allowed the pigs’ heads to reach the bowls and eat the rewards. On habituation days three and four, the pigs were individually guided to the experimental pen in pseudorandomized order and were allowed to explore for 5 min. The bowls were filled with only one piece of each reward (= six pieces). Habituation days five and six served to train the pigs to understand the connection between making a choice (= put the head through the opening) and obtaining the reward (= tray was pushed forward in the direction of the pig to make the rewards accessible). First, the tray was pulled back, which made the rewards inaccessible for the pig. Once a pig put its head, including its ears, through the opening, the tray was immediately pushed forward in the direction of the pig, giving access to the reward. As soon as the pig finished eating the rewards, the tray was pulled back again, and both bowls were refilled to start the next run. One session consisted of 12 runs, and the pig had 30 s to start eating the food in the bowl. This procedure was continued on habituation days seven, eight and nine, except for the fact that now a basic delay was introduced: the time until the tray was pushed forward to give access to the rewards was delayed by 2 s.

#### Preference test

The preference test was carried out on days seven, eight and nine of the habituation phase with one session per day and 15 runs per session, running in addition to the habituation session. The aim was to determine the most preferred food reward for each individual pig. Six different food rewards were used (see above). In each run, two spoons with two different food rewards were presented on the tray in close distance to one another in front of the right-hand side opening. Once presented, the pig was given 30 s to choose one of the rewards (= valid choice); otherwise, the spoons were removed (no choice). For the next run, the spoons were refilled with a new combination of food rewards after a break of 30 s (for more detailed information on the experimental procedure of the preference test, see ref.^20^). In each session, the order of the pigs was pseudorandomized with a different random order each session; every food combination was tested once per session in a randomized sequence, resulting in 15 runs per animal per session/day.

For each pig, the food item that was chosen the most frequently during the preference test was considered the preferred item and was used for the discrimination task and the delay-of-gratification task for this individual pig.

Three pigs were excluded from further experiments because they did not make a valid choice during the preference test, and no preferred food items could be identified.

#### Discrimination task

The discrimination task aimed to train the pigs to link two visual cues (a white or a black cup) with different reward sizes. The experimental procedure resembled the last 3 days of the habituation phase (days seven to nine), except that now a small reward (one piece) was presented in a bowl covered by a white cup and a larger reward (four pieces) in a bowl covered by a black cup. As soon as the pig made a choice (putting the head through an opening), the experimenter waited for 2 s (basis delay) until the tray was moved forward in the direction of the pig and the cup was removed, giving access to the chosen reward. If the pig did not make a choice after 30 s (no choice = omission), the bowls with the cups and food rewards were removed and refilled for the next run. One session per day was conducted, and each session started with four forced-choice runs where only one cup (white/black) with the respective reward was presented. The position (left/right) and order of the cups were pseudorandomized: both cups were presented twice (once on the right and once on the left). These forced-choice runs were conducted to ensure that the pig chose both cups and sides at the beginning of every session. Eight free-choice runs were then performed in which both cups were presented, and one choice was made for each run. Again, the position (left/right) of the black and white cups (large/small reward) was pseudorandomized, with a maximum of three identical stimulus presentations in a row. Subjects moved on to the delay-of-gratification task when the pig chose the large reward (black cup) at least seven times during the eight free-choice trials for one session. When a pig did not reach this learning criterion within 16 sessions, it was excluded from further experiments.

#### Delay-of-gratification task

The aim of the delay-of-gratification task was to determine if and for how long pigs would wait for a larger amount of their preferred reward instead of choosing a smaller amount of the reward immediately. The test consisted of one session per day. At the beginning of each session, two forced-choice runs were conducted (one left, one right, one white, and one black) followed by 10 free-choice runs. The procedure resembled the free-choice runs of the discrimination task, but now the time until the tray was pushed towards the opening after the pig made a choice was increasingly delayed for the larger reward from session to session (delay steps: 2 s, 4 s, 6 s, 10 s, 14 s, 20 s, 26 s, 32 s, and 40 s). After the run started, the pigs were given 30 s to make their choice. Three different behavioural reactions of the pigs were observed: first, the pig chose the black cup and waited until the end of the delay (large reward, delay accomplished). Second, the pig chose the white cup directly (small reward) or after choosing the black cup but not waiting to the end of the delay and rather switching to the position of the white cup (small reward, delay not accomplished). Third, the pig did not choose any cup within 30 s (omission). If a pig did not choose the black cup at least once within the 10 free-choice runs of a session, the experiment for this pig ended, and it was classified with the delay time from the previous session.

### Statistical analyses

The data analysis for this paper was generated using SAS software (version 9.4 of the SAS System for Windows, copyright 2002–2012; SAS Institute Inc. SAS and all other SAS Institute Inc. product or service names are registered trademarks or trademarks of SAS Institute Inc., Cary, NC, USA.) using the GLIMMIX procedure of SAS/STAT software. Binary data (reaching the learning criterion during the discrimination task (yes/no)) were evaluated with a logistic model, count data (number of sessions needed to reach the learning criterion during the discrimination task) were evaluated with a Poisson model, and data on the delay time during the delay-of-gratification task were analyzed by analysis of variance (ANOVA). The models contained the main effects replicate (1–2), age (younger vs. older) and their interaction. The mother ID was included as a random effect. Least squared means (LSMs) and their standard errors (SEs) were computed for each fixed effect in the model. Mean differences with a *p* < 0.05 were considered significant.

### Ethical note

The experimental procedure was approved by the ethics committee of the federal state of Mecklenburg-Western Pomerania, Germany (ref. no. 7221.3-2-010/17) and adhered to the legal requirements of the European Union (directive 2010/63/EU) and the ASAB/ABS guidelines for the use of animals in research. The study was carried out in compliance with the ARRIVE guidelines.

## Data Availability

The datasets generated during and/or analyzed during the current study are available from the corresponding author on reasonable request.
